# Improving the Understanding of the Immunopathogenesis of Lymphopenia as a Correlate of SARS-CoV-2 Infection Risk and Disease Progression in African Patients: Protocol for a Cross-sectional Study

**DOI:** 10.2196/21242

**Published:** 2021-03-04

**Authors:** Bamidele Abiodun Iwalokun, Adesola Olalekan, Eyitayo Adenipekun, Olabisi Ojo, Senapon Olusola Iwalokun, Bamidele Mutiu, Oluseyi Orija, Richard Adegbola, Babatunde Salako, Oluyemi Akinloye

**Affiliations:** 1 Nigerian Institute of Medical Research Lagos Nigeria; 2 Department of Medical Laboratory Sciences Faculty of Basic Medical Science, College of Medicine University of Lagos Lagos Nigeria; 3 Department of Natural Sciences Albany State University Georgia, GA United States; 4 Lagos State Ministry of Health Lagos Nigeria; 5 Department of Medical Microbiology & Parasitology College of Medicine Lagos State University Lagos Nigeria; 6 Department of Public Health National University San Diego, CA United States; 7 Microbiology Department Nigerian Institute of Medical Research Lagos Nigeria

**Keywords:** SARS-COV-2 infection, COVID-19, lymphopenia, immunopathogenesis, Nigeria

## Abstract

**Background:**

The COVID-19 pandemic, caused by SARS-CoV-2, continues to impact health systems throughout the world with serious medical challenges being imposed on many African countries like Nigeria. Although emerging studies have identified lymphopenia as a driver of cytokine storm, disease progression, and poor outcomes in infected patients, its immunopathogenesis, as well as environmental and genetic determinants, remain unclear. Understanding the interplay of these determinants in the context of lymphopenia and COVID-19 complications in patients in Africa may help with risk stratification and appropriate deployment of targeted treatment regimens with repurposed drugs to improve prognosis.

**Objective:**

This study is designed to investigate the role of vitamin D status, vasculopathy, apoptotic pathways, and vitamin D receptor (VDR) gene polymorphisms in the immunopathogenesis of lymphopenia among African people infected with SARS-CoV-2.

**Methods:**

This cross-sectional study will enroll 230 participants, categorized as “SARS-CoV-2 negative” (n=69), “COVID-19 mild” (n=32), “hospitalized” (n=92), and “recovered” (n=37), from two health facilities in Lagos, Nigeria. Sociodemographic data, travel history, and information on comorbidities will be obtained from case files and through a pretested, interview-based structured questionnaire. Venous blood samples (5 mL) collected between 8 AM and 10 AM and aliquoted into EDTA (ethylenediaminetetraacetic acid) and plain tubes will be used for complete blood count and CD4 T cell assays to determine lymphopenia (lymphocyte count <1000 cells/µL) and CD4 T lymphocyte levels, as well as to measure the concentrations of vitamin D, caspase 3, soluble vascular cell adhesion molecule-1 (sVCAM-1), and soluble Fas ligand (sFasL) using an autoanalyzer, flow cytometry, and ELISA (enzyme-linked immunosorbent assay) techniques. Genomic DNA will be extracted from the buffy coat and used as a template for the amplification of apoptosis-related genes (*Bax, Bcl-2, BCL2L12*) by polymerase chain reaction (PCR) and genotyping of VDR (Apa1, Fok1, and Bsm1) gene polymorphisms by the PCR restriction fragment length polymorphism method and capillary sequencing. Total RNA will also be extracted, reverse transcribed, and subsequently quantitated by reverse transcription PCR (RT-PCR) to monitor the expression of apoptosis genes in the four participant categories. Data analyses, which include a test of association between VDR gene polymorphisms and study outcomes (lymphopenia and hypovitaminosis D prevalence, mild/moderate and severe infections) will be performed using the R statistical software. Hardy-Weinberg equilibrium and linkage disequilibrium analyses for the alleles, genotypes, and haplotypes of the genotyped VDR gene will also be carried out.

**Results:**

A total of 45 participants comprising 37 SARS-CoV-2–negative and 8 COVID-19–recovered individuals have been enrolled so far. Their complete blood counts and CD4 T lymphocyte counts have been determined, and their serum samples and genomic DNA and RNA samples have been extracted and stored at –20 °C until further analyses. Other expected outcomes include the prevalence and distribution of lymphopenia and hypovitaminosis D in the control (SARS-CoV-2 negative), confirmed, hospitalized, and recovered SARS-CoV-2–positive participants; association of lymphopenia with CD4 T lymphocyte level, serum vitamin D, sVCAM-1, sFasL, and caspase 3 levels in hospitalized patients with COVID-19; expression levels of apoptosis-related genes among hospitalized participants with COVID-19, and those with lymphopenia compared to those without lymphopenia; and frequency distribution of the alleles, genotypes, and haplotypes of VDR gene polymorphisms in COVID-19–infected participants.

**Conclusions:**

This study will aid in the genotypic and phenotypic stratification of COVID-19–infected patients in Nigeria with and without lymphopenia to enable biomarker discovery and pave the way for the appropriate and timely deployment of patient-centered treatments to improve prognosis.

**International Registered Report Identifier (IRRID):**

DERR1-10.2196/21242

## Introduction

### Background

COVID-19, caused by the novel betacoronavirus SARS-CoV-2, which originated from Wuhan, China, in 2019, has spread to over 200 countries worldwide, infecting thousands of health care workers [1]. COVID-19 continues to impact health systems globally, with serious medical challenges imposed on many African countries such as Nigeria. The disease has contributed to an overwhelming number of hospitalizations of infected patients and rising needs for hospital beds and life-saving equipment [1-3]. COVID-19 incidence is increasing in many African countries due to burgeoning community tertiary transmission rates, which has resulted in a rising number of deaths and challenges in diagnosis and treatment [4]. As of May 12, 2020, in the African continent, a total of 69,126 cases and 2386 deaths, representing a case fatality rate of 3.5%, have been reported [5]. Nigeria currently ranks as the fourth most affected country in Africa, with 4787 cases, 158 deaths, and a case fatality rate of 3.3%, as of May 12, 2020. These indices are higher than the 356 cases and 19 deaths reported on April 22, 2020 [5]. Therefore, intensive research and innovation are required to provide new knowledge that can be utilized to improve supportive therapy; discover and develop new drugs, vaccines, and diagnostics; and initiate clinical trials in developed and developing countries around the world [3,5].

Although the need for ventilators and extracorporeal membrane oxygen for severe and critical cases of COVID-19 has been clarified [1,2,4], the immunopathogenesis of COVID-19 remains unclear [6]. However, emerging data from patients infected with COVID-19 indicate that genetic immunologic, metabolic, and environmental factors are involved in the pathogenesis of COVID-19 [6,7]. In addition to having no definitive treatment for COVID-19, there are also some supportive antiviral and corticosteroid treatments whose use remain controversial [8,9], suggesting that evidence-based supportive treatments are needed to combat COVID-19 globally. Clinically, patients with SAR-CoV-2 infection tend to experience mild symptoms such as fever, dry cough, anosmia, fatigue, dyspnea, headache, diarrhea, and sore throat, with up to 20% of these patients developing vascular and systemic complications such as leukocyte infiltration of the lungs, pneumonia, severe pneumonia, acute respiratory distress syndrome (ARDS), sepsis, and septic shock [2,10]. Recent studies in patients with COVID-19 have also shown lymphopenia to be a common immunosuppressive factor associated with a critical course of disease characterized by severe vascular abnormalities and poor outcomes [11-13]. Lymphopenia refers to lymphocyte count <1000-1500 cells per microliter of blood [14,15]. Currently, there is limited knowledge on how patients with COVID-19 develop lymphopenia, which incidentally can occur at an early stage of the disease [16].

### Association Between Lymphopenia, Leukocyte Apoptosis, Hypovitaminosis D, and Vasculitis

The fact that the biology of SARS-CoV-2 is closely related to SARS-CoV (severe acute respiratory syndrome–associated coronavirus), much can be learned from previous studies on patients infected with SARS. In the studies conducted by O’Donnell et al [17], Dancer et al [18], and Ding et al [19], lymphopenia was found to be associated with leukocyte apoptosis, low vitamin D status, and vascular dysfunction in Asian patients with SARS who exhibited different clinical manifestations. In Africa, leukocyte apoptosis has also been shown to play a role in the pathogenesis of several infectious diseases such as HIV, tuberculosis, and chronic obstructive pulmonary disease [20-22]. The FasL (Fas ligand)/Fas pathway, having caspace 3 as the key driver, represents the major leukocyte apoptosis pathway [23]. In addition, supporting leukocyte apoptosis is the upregulation in gene expression of agonists such as the *Bax* gene and downregulation of antagonists like the *Bcl-2* and *BCL2L12* genes [24]. Apart from regulating bone mineral homeostasis, vitamin D as the active metabolite 1,25-dihydroxyvitamin D3—abbreviated 1,25(OH)_2_ D3—is an important immune modulator in humans in disease and health [25]. Studies have further revealed vitamin D to downregulate the expression of the acute phase and TH1 inflammatory cytokines such as IFN-γ, IL-1, IL-6, and TNFβ in patients with respiratory diseases [26], indicating its immunosuppressive function. This also suggests that vitamin D may be beneficial in alleviating cytokine storm induced by lymphopenia and viremia in patients with COVID-19. Meanwhile, vitamin D deficiency has been shown to contribute to severe inflammatory response syndrome, sepsis, septic shock, and death [27,28], which are notable severe disease activities in patients with COVID-19. This finding also suggests that vitamin D supplementation in patients with COVID-19 may be beneficial clinically. This was actually evident in clinical trials reported by Batacchi et al [29] and Fanco et al [30] on the benefits of vitamin D supplementation in patients with chronic kidney and rheumatic diseases. The soluble vascular cell adhesion molecule-1 (svCAM-1) is a critical marker of vasculitis disorders such as diabetes, hypertension, cancer, systemic erythematosus, and rheumatoid arthritis. However, as a vasculitis disorder, the role of VCAM-1 in the pathogenesis of COVID-19 remains unknown [31,32].

### Vitamin D Receptor Gene Polymorphism and Serum Vitamin D Level

Genetic evidence has further implicated polymorphisms—Apa1 (rs7975232), Fok1 (rs2228570), and Bms1 (rs1544410)—in the vitamin D receptor (VDR) gene to be responsible for hypovitaminosis D, suggesting that host genetic factors may be the cause of hypovitaminosis D in humans, as previously reported by Santos et al [33]. Hypovitaminosis D in humans has been shown to occur despite adequate exposure to sunlight [34]. The VDR is a member of the nuclear receptor family whose gene is located on chromosome 9 with 9 exons in the human genome. Its three functional polymorphisms have been mapped to exon 2 of the second methionine start site, intron 8, and the three prime untranslated region of the gene [35-37]. The functional effects of these polymorphisms include changes in VDR protein folding and size, alterations in mRNA processing, splicing, and editing, as well as the binding affinity to the DNA response element of the adjacent gene under regulation [35-37].

### Rationale and Justification

To the best of our knowledge, there is a paucity of information on the burden of lymphopenia and its contribution to disease severity in African patients with COVID-19. No research efforts have been undertaken to investigate the pathophysiological relevance of hypovitaminosis D in the immunopathogenesis of lymphopenia coupled with the modulatory effects of VDR gene polymorphisms on the systemic level of vitamin D among SAR-CoV-2–negative, hospitalized patients with COVID-19 and survivors (ie, treated and recovered patients). Currently, like many African countries, vitamin D supplementation is not included in the treatment guidelines for hospitalized patients with COVID-19 in Nigeria, and the clinical benefits of vitamin D supplementation as a supportive treatment remain unknown. There is also limited understanding of the interactions between possible phenotypic and genotypic determinants of lymphopenia among Africans without SARS-CoV-2 infection or hospitalized due to COVID-19 and those who have recovered from the disease after successful treatment. Apart from the established contribution of comorbid factors such as diabetes, hypertension, hepatitis B and C, and HIV to the progression of SARS-CoV-2 infection with severe outcomes, the poor management of infected patients with repurposed drugs also contributes to poor prognosis. Therefore, an understanding of the interplay of the genetic and phenotypic determinants of the pathophysiological drivers of COVID-19 severity in African patients is needed. This study will aid in the stratification of COVID-19–infected Nigerian patients with and without lymphopenia phenotypically and genotypically to enable biomarker discovery and pave the way for the appropriate and timely deployment of patient-centered treatments with repurposed drugs to improve prognosis.

This pilot study aims to investigate the role of low vitamin D status, vasculopathy, apoptotic pathways, and VDR gene polymorphisms in the immunopathogenesis of lymphopenia among Nigerian patients infected with SARS-CoV-2.

### Specific Objectives

The study objectives are as follows:

1. Determine the prevalence and distribution of lymphopenia among Nigerian patients with COVID-19 at two selected health facilities in Lagos, Nigeria;
2. Examine serum vitamin D, FasL, and VCAM-1 levels in relation to the stages of COVID-19 infection among these patients compared to SARS-CoV-2–negative controls;
3. Evaluate the correlation of VDR gene polymorphism with serum vitamin D level and disease activity (mild symptoms, pneumonia, ARDS, and sepsis) of patients with COVID-19 who do or do not exhibit comorbidities;
4. Assess the level of expression of apoptotic agonist (*Bax*) and antagonists (*Bcl-2*, *BCL2L12*) in participants with and without SARS-CoV-2 infection;
5. Evaluate the relationship between lymphopenia and serum vitamin D, FasL, VCAM-1, and apoptotic markers in hospitalized patients with COVID-19.

### Hypotheses

Our hypotheses are as follows:

1. Lymphopenia is associated with an increased susceptibility to SARS-CoV-2 infection and COVID-19 severity with and without comorbidities;
2. Lymphopenia is associated with a low level of vitamin D, which is worsened by the VDR—Apa1 (rs7975232), Fok1 (rs2228570), and Bms1 (rs1544410)—gene polymorphisms in hospitalized patients with COVID-19;
3. Higher upregulation of the *Bax* gene and downregulation of the *Bcl-2* and *BCL2L12* genes occur in hospitalized patients with COVID-19 compared to recovered patients and controls (ie, no SAR-CoV-2 infection);
4. There is an inverse correlation of serum vitamin D with sVCAM-1, caspace 3, and soluble FasL (sFasL) levels in patients with SARS-CoV-2 infection, which does not exist in the controls.

## Methods

### Study Design

This quantitative study is cross-sectional in design. The participants will be recruited from the Nigerian Institute of Medical Research (NIMR) and the Infectious Disease Hospital (IDH) in Lagos. This study will be carried out over a period of 12 months (March 2020 to February 2021). It will involve questionnaire administration, hematological assays, flow cytometry, immunoassays, and molecular tests. The purpose of administering the questionnaire is to generate new data not reflected in the national epidemiological form for COVID-19 (such as history of/current treatment for tuberculosis; chronic obstructive pulmonary disease, and hospitalization in the previous 3 months prior to the onset of SARS-CoV-2–related symptoms) to improve the interpretation of test results and patients’ disease diagnosis (see Multimedia Appendix 1 for questionnaire).

SARS-CoV-2 diagnosis in patients will be based on a one-step real-time reverse transcription–polymerase chain reaction (rRT-PCR) test using nasopharyngeal swab samples at both study sites. Each swab sample will be collected in 3 mL of sterile viral transport medium (Rocky Mountain Biologicals, LLC). The rRT-PCR test per sample will be done by two independent, trained analysts whose results will be blinded from each other. Suspected patients whose rRT-PCR test reveals a negative result for 3 successive days using fresh nasopharyngeal samples and without progression of symptoms at presentation (to rule out false negative results) will be categorized as “SARS-CoV-2 negative.” Those who are SARS-CoV-2 positive but present mild symptoms will be called “COVID-19 mild.” Both categories of patients will be recruited at NIMR. The other two categories of patients—SARS-CoV-2–positive patients hospitalized due to complications (such as severe pneumonia, pulmonary edema, ARDS, pulmonary thrombosis, sepsis, and septic shock) and previously hospitalized COVID-19–confirmed (ie, rRT-PCR positive) patients who recovered from the disease—will be recruited at IDH. Some patients with COVID-19 who present mild or moderate symptoms will also be recruited as participants at IDH. Participant enrollment will be carried out consecutively until the desired sample size is acquired.

### Study Sites

The study will be conducted at NIMR and IDH. Established in 1977, NIMR is the leading national research institution in Nigeria, with the mandate to conduct basic, applied, translational, implementation, and clinical research on diseases of public health importance in the country. The NIMR Center for Human Virology and Genomics is one of the national SARS-CoV-2 diagnostics laboratories in the country handling preparedness and response to the COVID-19 pandemic in Nigeria. The center has rRT-PCR and Sanger sequencing facilities to detect and confirm SARS-CoV-2 diagnoses from nasopharyngeal and oropharyngeal swab samples. IDH is a specialist hospital involved in the diagnosis and treatment of patients with infectious diseases such as multidrug-resistant tuberculosis, HIV, and Lassa fever. Patients infected during the 2014 Ebola outbreak in Nigeria were also managed in this hospital. The hospital has a biosafety level 3 laboratory for emerging and re-emerging human and zoonotic pathogen containment in the country. In response to the current COVID-19 pandemic in Nigeria, IDH has also been given the responsibility of SARS-CoV-2 detection by rRT-PCR. In addition, the hospital has isolation and treatment centers with a good triage system to manage infected patients with mild, moderate, and severe manifestations of COVID-19. The hospital also has an intensive care unit with expert frontline health personnel such as cardiologists, pulmonary medicine physicians, and intensivists for the management of severe COVID-19 cases.

### Sample Size Determination and Study Population Grouping

The minimum sample size for the present study has been calculated based on the assumptions outlined below and using the following formula [38]:

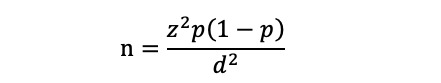



where *n* is the sample size, *z* represents the study confidence level for a two-tailed test at 1.96, *p* is the average prevalence rate of COVID-19 globally at 5.8% [1,2], and *d* is an error rate of 5%. By substituting the assumptions above into the formula, we obtained: n = (1.96^2^ × 0.058 × [1–0.058]) ÷ 0.05^2^ = 83.9. This was approximated to 84. This was followed by the addition of 10% to the calculated sample size to account for nonresponse and a design effect of 2.5 to provide a total sample size of 230 participants. The number of participants to be enrolled into the four groups of suspected and confirmed cases of COVID-19 was determined as follows:

- Group 1: participants who are SARS-CoV-2 negative at NIMR comprise 30% of the total sample size of 230 (n=69);
- Group 2: participants with SARS-CoV-2 infection who present mild or moderate symptoms of COVID-19 and are hospitalized at NIMR and IDH for isolation and treatment comprise 14% of 230 (n=32);
- Group 3: SARS-CoV-2 infected participants with severe manifestations of COVID-19 who are hospitalized at IDH comprise 40% of 230 (n=92);
- Group 4: previously hospitalized COVID-19–confirmed patients (rRT-PCR positive) who were treated at IDH and have recovered comprise 16% of 230 (n=37).

The percentage of the total sample size apportioned for each group of the participants was based on authors’ assumptions but also guided by the pattern of cases seen at the study sites (unpublished).

### Inclusion and Exclusion Criteria

For group 1 participants, the inclusion criteria are as follows: aged ≥15 years with or without selected comorbidities, and suspected of SARS-CoV-2 infection but tested negative by rRT-PCR.

For group 2 participants, the inclusion criteria are as follows: aged ≥15 years with or without selected comorbidities, with laboratory results indicative of SARS-CoV-2 infection by rRT-PCR and exhibition of mild or moderate symptoms, and admitted to the isolation center at IDH.

For group 3 participants, the inclusion criteria are as follows: aged ≥15 years with or without selected comorbidities, with laboratory results indicative of SARS-CoV-2 infection by rRT-PCR, and hospitalized in the treatment center for the management of the exhibited COVID-19 complications.

For group 4 participants, the inclusion criteria are as follows: aged ≥15 years with or without selected comorbidities, and previously hospitalized but recovered after treatment.

For all four groups, the exclusion criteria are as follows: incomplete metadata from the questionnaire and case report form, a diagnosis of cancer or end-stage renal disease, and declined consent by the individual to participate in the study.

The case definitions for these groups and definitions of selected comorbidities are provided in Textbox 1.

**Variables and definitions.**

**Participant groups**:

- SARS-CoV-2–negative patients: 3 successive negative test results by real-time reverse transcription–polymerase chain reaction (rRT-PCR) using a fresh nasopharyngeal swab sample per test and without progression of clinical symptoms elicited at presentation at the Nigerian Institute of Medical Research (NIMR)
- SARS-CoV-2–positive patients with mild or moderate symptoms of COVID-19: positive test results by rRT-PCR using a nasopharyngeal swab sample with at least two of the following symptoms: fever (axillary temperature >37.4 °C), dry cough, sore throat, anosmia, fatigue, myalgia, headache, vomiting, diarrhea, and pneumonia
- SARS-CoV-2–positive patients with COVID-19 complications: hospitalized patients with positive test results via rRT-PCR using nasopharyngeal swab sampling with at least two of the following complications: severe pneumonia, pulmonary edema, sepsis, septic shock, pulmonary thrombosis, and organ failure, as provided in the case report form by the attending physician coupled with the need for ventilation (mechanical or automated) or extracorporeal membrane oxygenation
- Recovered patients: previously hospitalized patients with confirmed COVID-19 (ie, rRT-PCR positive), with or without the selected comorbid factors, who have been discharged following successful treatment, as confirmed by 2 negative SARS-CoV-2 rRT-PCR test results 24 hours apart

**Selected comorbidities:**

- Hypertension: systolic blood pressure ≥140 mm Hg, diastolic blood pressure ≥90 mm Hg, or both, and on antihypertensive medication irrespective of blood pressure status
- Diabetes mellitus: fasting blood sugar ≥126 mg/dL or on medications for diabetes
- Tuberculosis: *Mycobacterium tuberculosis* culture positive, GeneExpert *M. tuberculosis* positive with or without rifampicin resistance using sputum sample or on medication for *M. tuberculosis* infection per national treatment guidelines
- Chronic obstructive pulmonary disease: exhibiting symptoms of a chronic cough, sputum production, difficult or labored breathing, and a spirometry FEV1 between 50%-79% of the predicted normal values and an FEV1/FVC ratio <70%
- Pneumonia: pulmonary disease with symptoms of fever, cough, chest pain, and rapid breathing >20 beats per minute at rest or shortness of breath
- Severe pneumonia: pneumonia characterized by hypoxemic respiratory failure, ventilation support, and sepsis
- HIV: HIV-1 seropositive or on medication for HIV/AIDS
- Hepatitis: on medications for hepatitis B or C
- Lymphopenia: total blood lymphocytes <1000-1500 cells per microliter of blood
- Vitamin D deficiency: values of 25(OH) D of ≤50 nmol/L (20 ng/mL)
- Vitamin D insufficiency: values of 25(OH) D ranging from 52 to 72 nmol/L (21-29 ng/mL)
- Hypovitaminosis D: values of 25(OH) D <75 nmol/L (30 ng/mL)
- Normal vitamin D status: values of 25(OH) D of ≥75 nmol/L (30 ng/mL) were considered sufficient for vitamin D

### Ethical Approval and Consent to Participate

This study has been submitted for approval to the NIMR Ethics Committee; approval for patient recruitment and access to sample collection at IDH has already been granted. Participants are required to give consent of participation in the study prior to enrollment. All data obtained from each study participant will be kept confidential and accessed only by the managing physician in charge for use in case management.

### Data Collection

Sociodemographic data (eg, age, gender, marital status, occupation, education), clinical data (diabetes, hypertension, hepatitis, HIV, need for ventilator or extracorporeal membrane oxygen, COVID-19 status), and travel history data (domestic and international travel to and from Lagos within 14 days of illness onset) of the participants will be collected using a structured questionnaire prepared in English. Anthropometric measurements of weight to the nearest 0.1 kg and height to the nearest 0.1 m will be performed using a meter rule and a digital weighing scale (Tanita Corp) with the patients bare footed and wearing light clothing. BMI will be computed with overweight and obesity classified as BMI ≥25 kg/m^2^ and BMI ≥30 kg/m^2^, respectively. The questionnaire will be pretested at the Lagos State University Teaching Hospital on 10 hospitalized patients to assess its comprehensibility and timeliness of administration with identified gaps corrected prior to data collection at the selected study sites. For severe and clinical cases, the attending clinician will assist and supervise data collection via a review of the case report form of the affected participants. Each participant’s questionnaire will be confirmed for completeness, and data coding will be done by the principal investigator prior to data entry into spreadsheets. Generally, the metadata collected from the participants will be blinded from analysts involved in SARS-CoV-2 infection confirmation by rRT-PCR.

### Laboratory Methods

#### Sample Collection and Processing

From every COVID-19–confirmed or treated case, an aliquot of 3 mL of venous blood obtained by venipuncture will be collected into an EDTA (ethylenediaminetetraacetic acid) vacutainer tube with 2 mL processed to obtain the buffy coat for molecular assays. Another aliquot of 3 mL venous blood will be collected in a plain bottle, allowed to clot, centrifuged at 2000 rpm for 10 minutes, and have its serum collected into a new bottle for serological and biochemical assays. Both sample types will be labeled using each study participant’s unique ID along with the date of collection, sample type, and study site. The processed samples will be stored at –80 °C prior to laboratory assays.

#### Blood Lymphocyte Count

A 0.5 mL aliquot of blood sample collected into the EDTA vacutainer tube will be used for white blood cells measurement. This will be done on fresh samples using the ADVIA 120 Hematology System. In the absence of a national surveillance range of lymphocyte counts in the Nigerian general population, a lymphocyte count less than 1000/μL will be considered as lymphopenia, as used by previous investigators in Nigeria and other parts of the world [14,15].

#### Measurement of Plasma sVCAM-1 and sFasL

The levels of sVCAM-1 and caspase 3 will be measured by an enzyme-linked immunosorbent assay (ELISA) kit (R&D Systems, Inc). sFasL levels were also measured by the ELISA kit (Bender Medsystems) in serum. The interassay variation coefficients of the two assay kits range from 2.5%-4.5%. Serum aliquot at 0.1 mL will be used for each individual ELISA as previously described [39,40].

#### Measurement of Serum 25(OH) D Concentrations

The serum 25(OH) D level will be measured by a competitive ELISA (kit provided by DRG International, Inc). Values of 25(OH) D ≤50 nmol/L (20 ng/mL) will be used to indicate vitamin D deficiency; values ranging from 52 to 72 nmol/L (21-29 ng/mL) will be considered as vitamin D insufficiency, and levels of ≥75 nmol/L (30 ng/mL) will be considered as sufficient levels of vitamin D [41].

#### DNA Extraction

Genomic DNA will be extracted from the buffy coat using the QIAamp DNA extraction kit (Qiagen) according to the manufacturer’s protocol. The extracted DNA will be suspended in TE buffer (pH 8.0) and stored at 4 °C prior to use. Isolated DNA concentration will be estimated using a nanodrop spectrophotometer at 260 nm and measured in ng/μL. Purity will be assessed based on an absorbance ratio of 260 nm to 280 nm (A_260nm_/A_280nm_) with values raging from 1.65-2.0 regarded as good quality.

#### RNA Extraction

One milliliter of EDTA blood will be used for total RNA extraction with the QIAamp RNA blood mini kit, following the procedure provided by the manufacturer (Qiagen). The spin column–bound RNA will be eluted with 50 mL of RNase-free water. To detect SARS-CoV-2, RNA will also be isolated from the 200 μL of nasopharyngeal swab samples using the Qiagen RNeasy mini extraction kit, following the manufacturer’s instructions (Qiagen). The spin column–bound RNA will be isolated with 50 mL of the provided elution buffer. Both RNA samples per participant will be stored at –80 °C. RNA yield will be estimated using a nanodrop spectrophotometer at 260 nm and measured in ng/μL. The isolated RNA solution with an A_260nm_/A_280nm_ value of 1.9-2.1 will be considered pure.

#### Gene Detection Assays

Both the apoptosis-related (*Bax, Bcl-2*, and *BCL2L12*) and VDR genes will be detected separately by monoplex polymerase chain reaction (PCR) with their specific primers, as shown in Table S1 in Multimedia Appendix 1, using the isolated genomic DNA as a template [42-44]. For each sample, the 20 μL reaction mixture will consist of a 5-fold dilution of the PCR master mix to give 1.5 mM MgCl_2_, 200 μΜ deoxynucleoside triphosphates (dNTPs), 1.25 U of Taq DNA polymerase and 1 × PCR buffer, pH 8.8, as a final composition (Isodyne), 10 picomoles of each forward and reverse primers, and 2 μL of genomic DNA template (approximately 100 ng). The final volume of the mixture will be adjusted to 20 μL by adding nuclease-free water. The PCR reaction will be performed on the SimpliAmpli Thermal Cycler (Thermo Fisher Scientific). The amplification condition for each reaction has also been provided in Table S1 in Multimedia Appendix 1. The PCR products derived from this procedure will be subjected to horizontal gel electrophoresis in 2% agarose gel prestained with ethidium bromide and a 100 bp DNA ladder marker for sizing. Both the visualization of the PCR bands and the sizing will be done under UV light using a photodocumentation gel dock (Thermo Fisher Scientific). Each PCR product will be diluted 10-fold and used as a template for real-time PCR.

#### RT-PCR

The production of first-strand complementary DNA (cDNA) from the RNA samples isolated from blood will be performed by the reverse transcription technique using oligo-dT primers. The 20 μL RT-PCR reaction will consist of Super Script II Reverse Transcriptase (Thermo Fisher Scientific, Invitrogen) and will be carried out according to the manufacturer’s protocol. The reaction will be performed on a heater block at 50 °C for 20 minutes. This will be followed by a 10-minute step at 99 °C to denature the enzyme, followed by cooling to 4 °C. The cDNA product will be stored at 4 °C and used for quantitative PCR (qPCR) within 24 hours of preparation.

#### Quantitative Real-Time PCR

The quantitative mRNA expression analysis of the apoptosis-related genes *Bax*, *Bcl-2*, and *BCL2L12* will be carried out using the real-time PCR method with SYBR Green 1 fluorescent dye [23,45]. The pair of primers designed for each of the genes under study can be seen in Table S1 in Multimedia Appendix 1. The primers for the apoptosis-related genes have been designed from the published reference sequence of each gene in the National Center for Biotechnology Information (NCBI) database using the Primer3Plus (eg, GenBank accession numbers NM_000633.2, ΝΜ_138761.3, ΝΜ_138639.1 and NM_002046.4 for BCL-2, BAX, BCL-x, and GAPDH, respectively). The *GAPDH* gene for glyceraldehyde 3-phosphate dehydrogenase was used as the internal control for all qPCR reactions. The qPCR reaction for each sample will be performed on an ABI Prism 7500 Thermal Cycler (Applied Biosystems) in duplicates, so as to address any data reproducibility issues. The 10 μL reaction mixture will consist of 0.5 μL of cDNA (for BAX, BCL2L12, and GAPDH) or a 10-fold diluted VDR PCR product, 50 picomoles of gene-specific primers, and 5 μL of Power SYBR Green I PCR master mix (Applied Biosystems, 4367659), containing AmpliTaq Gold DNA polymerase. The thermal conditions will consist of an initial denaturation and activation of a hot-start DNA polymerase step at 95 °C for 10 minutes, followed by 40 cycles of denaturation at 95 °C for 15 seconds and primer annealing and extension at 60 °C for 1 minute. After the quantitative amplification, a dissociation curve analysis will be performed to distinguish the amplified sequences of interest from any nonspecific ones or primer dimers via comparison of the melting temperatures of the formed PCR amplicons. The relative quantity of the mRNAs will be normalized relative to that of *GAPDH*, while the relative fold changes in the transcription level of these genes between asymptomatic and clinical cases, recovered and clinical cases, and asymptomatic and recovered cases will be calculated based on their cycle threshold (Ct) values using the comparative Ct method (2^–∆∆Ct^) [46]. qPCR amplification efficiency will be ascertained by performing a validation experiment as described by Kontanis et al [47] where amplification efficiency equals 10^–1/slope^.

#### One-Step rRT-PCR

For the diagnosis of SARS-CoV-2 infection, rRT-PCR will be carried out with RNA samples isolated from nasopharyngeal swabs as a template using the Viasure one-step rRT-PCR SARS-CoV-2 detection kit (CerTest Biotec). Briefly, 5 mL of isolated nasopharyngeal RNA, reconstituted SARS-CoV-2–positive control (noninfectious synthetic SARS-CoV-2 cDNA), or negative control will be added to each of the well strips containing specific primers and probes (targeting SARS-CoV-2 Orf1ab, N gene, and an internal control [IC]), buffer, dNTPs in a stabilized format prehydrated with 15 mL of supplied rehydration buffer. After a brief centrifugation, the one-step rRT-PCR will be done in a Bio-RadCFX96 Real-Time PCR Detection System (Bio-Rad) programmed as follows: 1 cycle of reverse transcription at 45 °C for 15 minutes, 1 cycle of initial denaturation at 95 °C for 2 minutes, followed by 45 cycles of denaturation at 95 °C for 10 seconds and an annealing/extension step at 60 °C for 50 seconds. Both positive and negative controls are included in each run to validate the reaction based on the absence of signals in the negative control well. Fluorogenic signals will be collected in the FAM (Orf1ab gene), ROX (N gene) and HEX (IC) channels of the CFX96 detection system. The signal generated by IC will be used to verify amplification. An rRT-PCR test for a sample will be regarded as positive if an amplification curve is generated for Orf1ab gene, N gene, and positive control with a Ct value less than 38 with or without an IC amplification curve. A presumptive test is indicated by the absence of the Orf1ab signal but presence of an amplification curve for the N gene and positive control with or without the IC amplification curve. A SARS-CoV-2 negative test will be indicated by the absence of an amplification curve for the Orf1ab gene and N gene but presence of amplification curves for the IC and positive control. All presumptive tests will be confirmed by repeating RNA isolation and pooling both RNA samples to increase RNA yield prior to rRT-PCR and combining epidemiological and clinical data for a SARS-CoV-2 diagnosis.

#### Genotyping of VDR Gene Polymorphisms

Genotyping for the Apa1 (rs7975232), Bsm1 (rs1544410), and Fok1 (rs2228570) polymorphisms in the VDR gene will be done using a PCR restriction fragment length polymorphism method as previously described [40-42]. Amplification was performed using a SimpliAmpli Thermal cycle (Thermo Fisher Scientific). The 20 μL reaction mixture will consist of a 5-fold dilution of PCR master mix to give 1.5 mM MgCl_2_, 200 μΜ dNTPs, 1.25 U of Taq DNA polymerase and 1 × PCR buffer, pH 8.8, as final composition (Isodyne), 10 picomoles of each forward and reverse primers, and 2 μL of genomic DNA template (approximately 100 ng). The final volume of the mixture will be adjusted to 20 μL by adding nuclease-free water. All PCR products will be digested with restriction enzymes (Fermentas–Euromedex), and the fragments will be separated by electrophoresis in a 3% agarose gel prestained with ethidium bromide (Table S1 in Multimedia Appendix 1). The Apa1, Bsm1, and Fok1 genotypes were defined by capital letters [42-44].

#### Capillary Sequencing of the VDR Gene and qPCR Amplicon

For quality control, a one-tenth of each of the VDR and qPCR amplicons will be randomly selected and cleaned using the EXOZAP reagent (Thermo Fisher Scientific). The cleaned amplicons will be sequenced at the Central Research Laboratory, a NIMR core sequencing facility, using a BigDye Terminator, version 3.1 (Thermo Fisher Scientific, Applied Biosystems), and appropriate primers (Table S1 in Multimedia Appendix 1). Each sequencing PCR reaction will contain 4 µL of the purified PCR product, 1.5 µL primer at 3.2 µM, and 2 µL of BigDye Terminator (Applied Biosystem). The PCR product will be further cleaned with HT ExoSAP. Finally, the contents of each well will be transferred to MicroAMP plates and then inserted in the Seqstudio for electrophoresis and data analysis (Thermo Fisher Scientific). The raw data obtained after electrophoresis will be stored as an *.abi file and then processed by base calling peaks of different colors representing adenine, cytosine, thymine, and guanine bases according to the fluorescence intensity of each of these peaks. For VDR gene, multiple sequence alignment will be done to confirm single nucleotide polymorphisms (SNPs) designed by Apa1, Bsm1, and Fok 1 in the PCR restriction fragment length polymorphism assays using the MEGA omega software. For the qPCR amplicons, the NCBI Basic Local Alignment Search Tool will be used to confirm the amplified SARS-CoV-2 N genes.

### Statistical Analysis

Coded data will be double entered into Microsoft Excel and Microsoft Access 2013 spreadsheets (Microsoft Corp). Data will be checked to ascertain the correctness and validity of each entry from the questionnaire before transfer for analysis. The entered data will be password protected and each record per participant will be given a unique identifier. Statistical analysis will be performed using SPSS (version 22.0, IBM Corp).

Fold changes in the expression levels of apoptosis-related genes (*Bax, Bcl-2, BCL2L12)* will be illustrated with a bar graph. Categorical data such as lymphopenia, gender, age group, and prevalence of hypovitaminosis D will be summarized as counts and percentages and analyzed by the chi-square test. The Kolmogorov-Smirnov test will be performed to confirm the normality of variables. The assumed continuous and normally distributed variables such as VCAM-1, FasL, serum vitamin D, and caspase 3 levels will be summarized as mean and standard error of mean and analyzed using the Student *t* test (2 categories of participants) and one-way analysis of variance (ANOVA) (3 or 4 categories of participants). For variables whose data are not normally distributed, the median will be computed. For such variables, comparison between two groups will be performed using the Mann-Whitney *U* test, while the Kruskal-Wallis test will be performed to compare 3 or 4 categories of participants. Analyses will be further stratified by comorbid factors such as diabetes, hypertension, HIV, obesity, chronic obstructive pulmonary disease , asthma, and hepatitis to reduce bias. These tests will also be used to evaluate the association between VDR polymorphisms, serum vitamin D, sVCAM-1, and FasL levels. Logistic regressions with odd ratios and 95% CIs calculated via a recessive model will be used to test the association of each of the VDR SNPs with COVID-19 severity among hospitalized participants. Pearson correlation coefficient analysis will be used to test the correlation of lymphopenia with vitamin D, sVCAM-1, sFasL, and caspase 3 levels. The Hardy Weinberg equilibrium principle will be used to examine the distribution of VDR alleles and genotypes for each of the studied SNPs. The haplotype frequency of the 3 VDR functional SNPs will also be compared across participant groups using the chi-square test and have their linkage disequilibrium determined. Outcome of analyses with *P*<.05 will be considered significant.

## Results

A total of 45 participants comprising 37 SARS-CoV-2–negative and 8 COVID-19–recovered individuals have been enrolled so far; their complete blood counts and CD4 T lymphocyte counts have been determined, and their serum samples and genomic DNA and RNA samples have been extracted and stored at –20 °C until further analyses. The study is still in the recruitment phase, which is expected to be completed in November 2020. However, immunological and molecular assays of RNA and DNA samples already isolated will be done in parallel.

## Discussion

The prevalence of COVID-19 and its associated adverse outcomes and economic constraints in Nigeria continue to increase. Therefore, it is important to understand the role of lymphopenia in the clinical outcome of SARS-CoV-2 infection and its underlying mechanisms. There is evidence that leukocyte apoptosis could contribute to lymphopenia and, together with hypovitaminosis D and vasculopathy, result in severe manifestations of the previous human coronavirus pandemics caused by SARS and the Middle East respiratory syndrome. However, the contributions of these pathophysiological factors to SARS-CoV-2 infection susceptibility and COVID-19 severity, especially among people in Africa, remain unclear. This information is very important for guiding an understanding of the immunopathogenesis of lymphopenia based on the influence of environmental, metabolic, apoptotic, and genetic factors among Nigerians. This translational research approach will further help in stratifying Nigerians infected with SARS-CoV-2 for treatment with an appropriate and timely repurposed drug regimen to improve prognosis. Discovering the role of VDR polymorphism in the occurrence of vitamin D deficiency and insufficiency among patients with SARS-CoV-2 infection will provide insight into how the clinical benefits of vitamin D supplementation can be optimized for those infected and/or hospitalized due to COVID-19 to reduce mortality, accelerate recovery from the illness, and promote health among people in Nigeria. This study will also explore the potential deployment of VDR genotyping and apoptosis-related immunological and molecular assays as components of the laboratory work-up of SARS-CoV-2–infected Nigerians to further improve the quality of care of patients with COVID-19 in the country.

## Appendix

Multimedia Appendix 1 Supplementary materials.

## Abbreviations

ANOVA: analysis of variance
ARDS: acute respiratory distress syndrome
cDNA: complementary DNA
Ct: cycle threshold
dNTP: deoxynucleoside triphosphate
EDTA: ethylenediaminetetraacetic acid
ELISA: enzyme-linked immunosorbent assay
FasL: Fas ligand
IC: internal control
IDH: Infectious Disease Hospital
NCBI: National Center for Biotechnology Information
NIMR: Nigerian Institute of Medical Research
PCR: polymerase chain reaction
qPCR: quantitative polymerase chain reaction
rRT-PCR: real-time reverse transcription–polymerase chain reaction
RT-PCR: reverse transcription–polymerase chain reaction
SARS: severe acute respiratory syndrome
SARS-CoV: severe acute respiratory syndrome–associated coronavirus
sFasL: soluble Fas ligand
SNP: single nucleotide polymorphism
sVCAM-1: soluble vascular cell adhesion molecule-1
VCAM-1: vascular cell adhesion molecule-1
VDR: vitamin D receptor
