# Supplementary material for: Improving the Understanding of the Immunopathogenesis of Lymphopenia as a Correlate of SARS-CoV-2 Infection Risk and Disease Progression in African Patients: Protocol for a Cross-sectional Study
Source: JMIR Res Protoc. 2021 Mar 4;10(3):e21242. doi: 10.2196/21242 (PMC7935252; doi:10.2196/21242)
Supplement: Multimedia Appendix 1 [file resprot_v10i3e21242_app1.docx]

**Table 1 List of primers to be used for PCR in this study**

| **S/N** | ***Gene/SNP** | **Primer Sequence** | **Expected Size** | **Amplification**  **Conditions** | **Reference** |
| --- | --- | --- | --- | --- | --- |
| 1 | BCL-2 | F: 5′-TTTGAGTTCG GTGGGGTCAT-3  R: 5′-TGACTTCACT TGTGGCCCAG-3′ | 275 bp | 95^0^C 5 min  95^0^C 30 s  58^0^C 30 s  72^0^C 1 min  72^0^C 5 min | 43 |
| 2 | BAX | F: 5′-TGGCAGCTGA CATGTTTTCT GAC-3′  R : 5′-TCACCCAACC ACCCTGGTCT T-3′ | 195 bp | 95^0^C 5 min  95^0^C 30 s  60^0^C 45 s  72^0^C 1 min  72^0^C 7 min | 43 |
| 3 | BCL2LI2 | F: 5′-CCCTCGGCCT TGCTCTCT-3′  R: 5′-TCCGCAGTAT GGCTTCCTTC-3′ | 182 bp | 95^0^C 5 min  95^0^C 30 s  56^0^C 30 s  72^0^C 1 min  72^0^C 10 min | 43 |
| 4 | GAPDH | F : 5′-CCTCCCGCTT CGCTCTCT-3′  R : 5′-CCGTTGACTC CGACCTTCAC-3′ | 116 bp | 95^0^C 5 min  95^0^C 30 s  57^0^C 30 s  72^0^C 1 min  72^0^C 10 min | 43 |
| 5 | VDR (Apa1) | F: 5’-GTCGCTGAGGGATGG-3’  R : 5’-GTCGGCTAGCTTCTGGAT-3’ | 400 bp  A = 400 *(Apa1*)  a = 232 + 168 (Apa1) | 95^0^C 5 min  95^0^C 1 min  58^0^C 1 min  72^0^C 1 min  72^0^C 10 min | 40 |
| 6 | VDR (Bsm1) | F :5’-CAACCAAGACTACAAGTACCGCGTCAGTGA-3’  R : -AACCAGCGGGAAGAGGTCAAGGG-3’ | 825 bp  B,= 825 (Bsm1)  b = 650 + 175 (Bsm1) | 94^0^C 4 min  94^0^C 30 s  63^0^C 30 s  72^0^C 1 min  72^0^C 5 min | 41 |
| 7 | VDR (Fok1) | F : AGCTGGCCCTGGCACTGACTCTGCTCT-3’  R : ATGGAAACACCTTGCTTGCTTCTCCCTC-3’ | 265 bp  F = 265 (Fok1)  f = 169 + 96 (Fok1) | 94^0^C 5 min  94^0^C 30 s  61^0^C 30 s  72^0^C 1 min  72^0^C 10 min | 42 |

Amplification for ApaI is 30 cycles, for BsmI is 35 cycles, for FokI is 35 cycles and for *Bcl-2, Bax, BCL2LI2 and GAPDH* is 40 cycles. **Bcl-2, Bax, BCL2LI2 and GAPDH* gene expression was also measured by RT-PCR with amplification conditions indicated in the body of the protocol

**COVID-19 STUDY QUESTIONNAIRE**

**TITLE: COVID-19: Improving the Understanding of ImmunopathoGenesis of LYmphopenia as a Correlate of SARS-COV-2 Infection Risk and disease Progression in African Patients: UGLY SARS-COV-2 Study**

| Health Facility:_______________State:___________ LGA:______________ Ward:_________ | |
| --- | --- |
| **1. Current Status** | |
| Asymptomatic [ ] Mild/Moderate [ ] Severe [ ] Recovered [ ] alive [ ] Dead [ ] | |
| At Home [ ] Isolation Centre [ ] Treatment Centre [ ] ICU [ ] | |
| **2. Case Identifier Information** | |
| Age: 10– 20 [ ] 20-30 [ ] 31–40 [ ] 41–50[ ] 51–60 [ ] 61 – 70[ ] 71-80 [ ] >80 [ ] Specify____  Date of Birth< DD/MM/YY ___/___/___ | |
| Gender: Male [ ] Female [ ] | |
| Marital Status: Single [ ] Married [ ] Divorced [ ] Separated [ ] Widowed [ ] Others: ______ | |
| Level of Education: None[ ] Primary [ ] Secondary[ ] OND/NCE[ ] HND[ ] Bsc[ ] MSc [ ] PhD [ ] | |
| Religion: Christianity [ ] Islam [ ] traditional Religion [ ] Others Pls specify____________________ | |
| Ethnicity: Yoruba [ ] Ibo [ ] Hausa [ ] Others Pls Specify______________________ | |
| Occupation: Unemployed [ ] Housewife [ ] Student [ ] Trading [ ] Artisan [ ] Civil Servnnt [ ] Private [ ] Retired [ ] Others Pls Specify_____________________________ | |
| Case Identification: Suspected ; [ ] Probable [ ] Confirmed [ ] | |
| **3. Patient’s home caregiver respondent Identification** | |
| Gender | |
| Date of Birth: DD/MM/YY ___/___/____ | |
| Relationship to Patient: Father [ ] Mother [ ] Uncle [ ] Aunt [ ] Brother [ ] Sister [ ] Others__________ | |
| **4. Patient symptoms from Disease Onset** | |
| Data of first symptom onset: DD/MM/YY ___/__/__ 1 -5days ago [ ] 6-10 days ago, Specify_______ | |
| Fever (Axillary temp > 37.4^0^C) or History of Fever: Yes [ ] No [ ] Unknown [ ] | |
| Shortness of Breadth Yes [ ] No [ ] Unknown [ ] | |
| Sore Throat Yes [ ] No [ ] Unknown [ ] | |
| Headache Yes [ ] No [ ] Unknown [ ] | |
| Fatigue Yes [ ] No [ ] Unknown [ ] | |
| Dry cough Yes [ ] No [ ] Unknown [ ] | |
| Diarrhea Yes [ ] No [ ] Unknown [ ] | |
| Loss of sense of smell Yes [ ] No [ ] Unknown [ ] | |
| Loss of sense of taste Yes [ ] No [ ] Unknown [ ] | |
| Body pain Yes [ ] No [ ] Unknown [ ] | |
| Running Nose Yes [ ] No [ ] Unknown [ ] | |
| Nasal congestion Yes [ ] No [ ] Unknown [ ] | |
| Nausea Yes [ ] No [ ] Unknown [ ] | |
| Vomiting Yes [ ] No [ ] Unknown [ ] | |
| Abdominal pain Yes [ ] No [ ] Unknown [ ] | |
| **5. Anthropometric Measurement** | |
| Body weight [ ] kg (nearest 0.1 kg) Height [ ] (nearest 0.1 m) BMI body weight / Height^2^ [ ] | |
| **6. Sample Collection Information** | |
| Date of collection DD/MM/YY __/__/__ Nasal swab [ ] throat swab [ ] Nasopharyngeal [ ] Others _______________________________ | |
| Date of collection DD/MM/YY __/__/__ Venous Blood [ ] CSF [ ] Others__________________ | |
| **7. Co-morbidity Information** | |
| Obesity Yes [ ] No [ ] Unknown [ ] | |
| Diabetes Yes [ ] No [ ] Unknown [ ] | |
| Hypertension Yes [ ] No [ ] Unknown [ ] | |
| Hepatitis B Yes [ ] No [ ] Unknown [ ] | |
| Hepatitis C Yes [ ] No [ ] Unknown [ ] | |
| HIV Yes [ ] No [ ] Unknown [ ] | |
| Heart problem Yes [ ] No [ ] Unknown [ ] | |
| Asthma Yes [ ] No [ ] Unknown [ ] | |
| Chronic Obstructive pulmonary disease (COPD) Yes [ ] No [ ] Unknown [ ] | |
| Cancer Yes [ ] No [ ] Unknown [ ] | |
| TB Yes [ ] No [ ] Unknown [ ] | |
| Sickle cell Disease Yes [ ] No [ ] Unknown [ ] | |
| Others Pls specificy____________________________________________________ | |
| **8. Current disease associated condition and healthcare needs** | |
| Pneumonia Yes [ ] No [ ] Unknown [ ] | |
| Acute Respiratory Distress Syndrome Yes [ ] No [ ] Unknown [ ] | |
| Sever Acute Respiratory Distress Syndrome Yes [ ] No [ ] Unknown [ ] | |
| Sepsis Yes [ ] No [ ] Unknown [ ] | |
| Septic Shock Yes [ ] No [ ] Unknown [ ] | |
| Gangrene in the Extremities Yes [ ] No [ ] Unknown [ ] | |
| Required Hospitalisation Yes [ ] No [ ] Unknown [ ] | |
| Ventilation Required Yes [ ] No [ ] Unknown [ ] | |
| Extracoporeal Membrane Oxygen required Yes [ ] No [ ] Unknown [ ] | |
| ICU care required Yes [ ] No [ ] Unknown [ ] | |
| **9. Human exposure in the 14 days prior to illness onset** | |
| Have you traveled out of your home domestically in the last 14 days? | Yes [ ] No [ ] Not sure [ ]  If Yes, date of travel: dd/mm/yy  __/__/___  State:_________________  LGA:_________________  Ward:_________________ |
| Have you traveled out of your home internationally in the last 14 days? | Yes [ ] No [ ] Not sure [ ]  If Yes, date of travel: dd/mm/yy  __/__/___  Country:_________________  City:___________________  Hotel:___________________  House Address __________________ |
| Have been to a mass gathering recently? | Yes [ ] No [ ] Not sure [ ] |
| Have you been with a COVID-19 suspected or confirmed patient recently? | Yes [ ] No [ ] Not sure [ ] |
| Have you been to a health facility where COVI-19 suspected or confirmed patients are cared for? | Yes [ ] No [ ] Not sure [ ] |
| Have you ever had BCG vaccination before? | Have you been to a health facility where COVI-19 suspected or confirmed patients are cared for? |
| **10. Form Completion Status** | |
| Yes [ ] No [ ] Partially [ ] | |
| Reasons for No or Partial form completion | |
| Missed [ ] Not Attempted [ ] Refusal [ ] Others Pls specify | |
| **THANK YOU** | |
